# Difficult Decisions: A Qualitative Exploration of the Statistical Decision Making Process from the Perspectives of Psychology Students and Academics

**DOI:** 10.3389/fpsyg.2016.00188

**Published:** 2016-02-16

**Authors:** Peter J. Allen, Kate P. Dorozenko, Lynne D. Roberts

**Affiliations:** ^1^School of Psychology and Speech Pathology, Curtin UniversityPerth, WA, Australia; ^2^School of Occupational Therapy and Social Work, Curtin UniversityPerth, WA, Australia

**Keywords:** statistics, research methods, decision making, selection skills, StatHand, decision tree, graphic organizer, teaching and learning

## Abstract

Quantitative research methods are essential to the development of professional competence in psychology. They are also an area of weakness for many students. In particular, students are known to struggle with the skill of selecting quantitative analytical strategies appropriate for common research questions, hypotheses and data types. To begin understanding this apparent deficit, we presented nine psychology undergraduates (who had all completed at least one quantitative methods course) with brief research vignettes, and asked them to explicate the process they would follow to identify an appropriate statistical technique for each. Thematic analysis revealed that all participants found this task challenging, and even those who had completed several research methods courses struggled to articulate how they would approach the vignettes on more than a very superficial and intuitive level. While some students recognized that there is a systematic decision making process that can be followed, none could describe it clearly or completely. We then presented the same vignettes to 10 psychology academics with particular expertise in conducting research and/or research methods instruction. Predictably, these “experts” were able to describe a far more systematic, comprehensive, flexible, and nuanced approach to statistical decision making, which begins early in the research process, and pays consideration to multiple contextual factors. They were sensitive to the challenges that students experience when making statistical decisions, which they attributed partially to how research methods and statistics are commonly taught. This sensitivity was reflected in their pedagogic practices. When asked to consider the format and features of an aid that could facilitate the statistical decision making process, both groups expressed a preference for an accessible, comprehensive and reputable resource that follows a basic decision tree logic. For the academics in particular, this aid should function as a teaching tool, which engages the user with each choice-point in the decision making process, rather than simply providing an “answer.” Based on these findings, we offer suggestions for tools and strategies that could be deployed in the research methods classroom to facilitate and strengthen students' statistical decision making abilities.

## Introduction

Quantitative research methods have played a central role in the progress of modern psychology (Benjamin, 2014), and a knowledge of quantitative methods is recognized as essential to the development of psychological literacy (McGovern et al., 2010) and the professional competence of psychology graduates. These points are reflected in the core competencies and graduate attributes specified by accrediting agencies worldwide (e.g., American Psychological Association Board of Educational Affairs Task Force on Psychology Major Competencies, 2013; Australian Psychology Accreditation Council, 2014; British Psychological Society, 2015), and by the prominent position that quantitative methods hold in undergraduate psychology curricula (Perlman and McCann, 1999). This prominence reflects a widely held understanding that an ability to critically evaluate relevant research literature, the vast majority of which is quantitative (Kidd, 2002), is a necessary precursor to evidence-based practice (American Psychological Association Presidential Task Force on Evidence Based Practice, 2006). Engaging students regularly in all aspects of the research process is recognized as fundamental to teaching quantitative methods successfully (Bradstreet, 1996; Stoloff et al., 2015), hence the typical undergraduate psychology degree provides students with multiple opportunities to conduct empirical research, either individually or in collaboration with others (Perlman and McCann, 2005).

### Selecting appropriate statistics

Despite their prominence and utility, quantitative research methods, and particularly statistics, are known areas of weakness for many psychology students (Garfield and Ben-Zvi, 2007; Murtonen et al., 2008). Students are known to particularly struggle with the development of “selection skills” (Ware and Chastain, 1989, p. 222), or the selection of appropriate statistical tests and procedures for different types of research questions, hypotheses and data types. For example, when Gardner and Hudson (1999) asked students to identify appropriate statistical analyses for a series of brief research vignettes, most found the task extremely difficult, and performed poorly. Even though most had completed at least six research methods and statistics units^1^, they managed to identify appropriate statistics for just 25.3% of the scenarios. Gardner and Hudson coded an additional 15.7% of the students' answers as “partially correct.” When the researchers questioned the students about how they made their decisions, several explanations for the poor performance emerged. These explanations included students misinterpreting the research scenarios, being unable to actually name known procedures, misidentifying variables' levels of measurement, and answering based on misleading key words and tables of data (which were formatted horizontally rather than vertically, as they would typically appear in a spreadsheet).

If students are required to simply recognize, rather than recall appropriate statistics, their performance is similarly limited. For example, Ware and Chastain (1989) developed a short multiple-choice selection skill test containing questions pitched at a level they believed a typical student would be able to answer on completion of an introductory statistics unit. However, when they gave the test to students at the conclusion of such a unit, the students answered fewer than 45% of the items correctly. The researchers attributed this poor performance, at least partially, to a curriculum that presented statistical techniques “one at a time” (p. 226), and provided students with few opportunities to practice selection skills. Several other researchers have made similar observations, noting that the typical research methods and statistics unit places far greater emphasis on using known statistical techniques than it does on exploring the circumstances in which they are appropriate (e.g., Bradstreet, 1996; Quilici and Mayer, 1996, 2002; Lovett and Greenhouse, 2000; Yan and Lavigne, 2014). In other words, the difficulties that students experience when placed in situations where they must work out *which technique* to use may be simply attributable to a lack of practice.

When students are provided with opportunities to practice their selection skills, performance increases somewhat (e.g., Ware and Chastain, 1991). For example, when Quilici and Mayer (2002) trained students to focus on the structural features of research scenarios (e.g., the nature of the independent and dependent variables, and the relationship between them), rather than their surface-level characteristics (e.g., the topic of the research), their ability to correctly categorize basic scenarios according to how they would be analyzed improved. The training also improved students' abilities to produce new scenarios with the same structural features as existing ones. However, performance was still far from perfect on both outcome measures. More recently, similar findings were reported by Yan and Lavigne (2014), who also focused their training and categorization tasks on just three basic statistical tests (i.e., independent samples *t*-test, chi-square test of contingencies, and Pearson's product moment correlation coefficient).

These findings suggest that selection skills are underpinned by a “structural awareness” (Quilici and Mayer, 2002, p. 326), which reflects an ability to disregard the surface features of a research scenario, and instead focus on its structural features and the relations between them. Consider the following section of research vignette four, presented in Appendix A in Supplementary Material:

*You work at a university library, and have been tasked with finding out which students accrue the largest ‘overdue fines’. The head librarian has provided you with a data file that gives you the total amount of fines (in dollars) accrued by each borrower during the previous 12 months, along with a range of additional information (e.g., each borrower's course of study, age, gender, number of items borrowed etc.)*.

Identifying an appropriate statistical technique for this scenario requires disregarding its “cover story” or surface-level features, and focusing on identifying its structural features and the relationships between them. In this case, it requires firstly recognizing that the broad intent is prediction (rather than, for example, a comparison between means) and identifying the independent and dependent variables. Here, there are several independent variables of varying types (i.e., dichotomous, nominal, and continuous), and one continuous dependent variable. It secondly involves constructing a generic conceptual model in which the relationships between structural features are represented. In this instance, the intent of the researcher is to use a combination of several independent variables to predict scores on a continuous dependent variable. Finally, it requires integrating the conceptual model with existing knowledge to find possible solutions. For many research scenarios there are a range of statistical techniques that could be used to analyze the data, requiring the researcher to compare possible techniques to determine the most appropriate statistical technique for the particular set of circumstances. While sometimes there may be two or more equally suitable techniques, here the most obvious solution is multiple linear regression, which would provide coefficients useful for addressing the head librarian's question, although additional considerations (e.g., the likely distribution of the dependent variable) may suggest other possibilities. An iterative process may be required between statistical technique selection and testing of assumptions in order to make the final decision.

Without assistance, students find the process described above very challenging. However, “experts” do not. While the point of transition from novice to expert in this specific context is not known, it appears to necessitate a substantial amount of experience. For example, Rabinowitz and Hogan (2008) recruited graduate students enrolled in Masters and PhD courses at a university with “a very well established psychometrics program” (p. 401) to complete a series of triad judgment tasks. In these tasks they were required to identify which of two statistics scenarios “goes best” with a specified target scenario. When faced with the option of selecting a scenario that shared structural but not surface characteristics with the target, or the reverse, even those participants with the greatest amount of experience (i.e., those who had completed between four and eight statistics units previously) did not reliably choose on the basis of structure. Those with the least experience chose based on surface characteristics. Indeed, it was not until the choice was between a scenario that was similar on structural characteristics only and one that was dissimilar on both structure and surface that these “experienced” participants reliably chose based on the structural features of the scenarios. Furthermore, in the Gardner and Hudson (1999) study described earlier, even the most experienced members of their sample (students admitted entry into fourth year, Masters and PhD courses in psychology and education) rarely answered more than 50% of the scenarios they were exposed to correctly.

Beyond the focus on surface and structural components of research scenarios, little is known about how students and experts select statistical tests. The first aim of this research was to develop a rich account of the strategies that psychology students and psychology academics (with expertise in research and/or research methods instruction) use to decide which statistical tests and procedures are appropriate for different research questions, hypotheses and data types.

### Decision making aids

The preceding section suggests several points. First, even experienced students are not able to autonomously select appropriate statistics in a reliable way. Second, students are often required to make such decisions relatively early in their courses, but are not always explicitly taught how to make them. Third, making such decisions incorrectly can carry substantial negative consequences. At a very pragmatic level, basing a research report on the results of the “wrong” statistical test, will lead to incorrect interpretations and likely poor grades. At a deeper level, it reveals deficits in statistical reasoning or thinking (Bradstreet, 1996; Chance, 2002). Collectively, these points suggest a need for aids or resources that students can rely on to facilitate the statistical decision making process, and perhaps also speed their transition from novice to autonomous expert.

Numerous such aids have been developed, including tip sheets which sort statistical tests according to their defining characteristics (e.g., Twycross and Shields, 2004), and charts which link common research goals to corresponding statistics (e.g., Beitz, 1998). However, the aids which have gained most traction are based around the idea of a “decision tree” or “graphic organizer.” Such resources facilitate the decision making process by prompting the user to engage with each structural feature of their research design, as well as the hierarchical and vertical relationships between them (Schau and Mattern, 1997). In the short term, this ensures that the user considers all relevant aspects of the design before deciding on a statistical test, thus increasing the likelihood that a correct decision will ultimately be made. In the longer term, decision trees help users integrate their knowledge of statistical concepts into coherent and organized schemata, which can be quickly and effectively activated when required (Yin, 2012).

Graphic organizers to guide statistical decision making have been used for at least half a century (e.g., Siegel, 1956; Mock, 1972), and are now commonly included in statistics textbooks (e.g., Field, 2013; Tabachnick and Fidell, 2013; Allen et al., 2014). Their inclusion in such books is supported empirically by research on the efficacy graphic organizers generally (e.g., Nesbit and Adesope, 2006) and in the context of statistical decision making specifically. For example, Carlson and colleagues (Carlson et al., 2005; Protsman and Carlson, 2008) demonstrated that graphic organizers could facilitate significantly faster and more accurate (by a multiple of three) statistical decision making, compared to more traditional methods of statistical test selection (e.g., by searching through a familiar textbook). The graphic organizer method was also significantly more popular than the textbook method amongst students.

Regardless of their popularity, traditional statistics decision trees also have a number of limitations. For example, they are often constrained by the requirement that they fit within the pages of a textbook, and when given to students without accompanying resources (e.g., definitions of key terms) they can be of limited use. Koch and Gobell (1999) attempted to overcome this limitation by translating and elaborating a paper-based decision tree for delivery on the world-wide-web. In doing so, they were able to provide students with a range of additional resources, including definitions and information about how to run and interpret the tests that their online decision tree helped students identify. Like Carlson and colleagues (Carlson et al., 2005; Protsman and Carlson, 2008), Koch and Gobell found that students using their decision tree were better able to identify appropriate statistical tests than students in a comparison condition. Unfortunately, Koch and Gobell's website is no longer active, and many of the online statistical decision trees currently available are of dubious quality or offer little more than could be contained within a traditional paper decision tree.

Aids or resources developed for students to facilitate the statistical decision making process are most likely to be promoted by instructors (experts) and adopted by students if they are developed with expressed needs and preferences of both stakeholder groups in mind. We could locate no research that asked about such needs and preferences regarding statistical decision making aids. Therefore, the second aim of our study was to elicit students' and academics' views on the nature of resources that could facilitate the statistical decision making process.

### The current study

As noted previously, the two key aims of the current study were to (a) develop a rich account of the strategies that psychology students and psychology academics (with expertise in research and/or research methods instruction) use to decide which statistical tests and procedures are appropriate for different research questions, hypotheses and data types; and (b) elicit students' and academics' views on the nature of resources that could facilitate the statistical decision making process. The study was conducted in two phases. In phase one, undergraduate psychology students were engaged in semi-structured interviews centered on the role and value of statistics, the process of statistical test selection, and the possible characteristics of aids which may facilitate this process. The interpretations from phase one informed the development of phase two. In phase two, psychology academics were engaged in similar interviews, which also queried their perspectives on the challenges students experience when choosing between statistical tests. The findings from both phases will be integrated in the discussion.

This research complies with the guidelines for the conduct of research involving human participants, as published by the Australian National Health and Medical Research Council (National Health and Medical Research Council, Australian Research Council and Australian Vice-Chancellors' Committee, 2007). Prior to recruitment of participants, the study was reviewed and approved by the Human Research Ethics Committee at Curtin University.

## Phase one: Students' decision making

### Methods

#### Participants

The phase one participants were nine undergraduate psychology students (five female) with a mean age of 22 years. All had recently completed one or more quantitative research methods and statistics units (median = 3; range = 1–5) and were, on average, in their third year of study. During the interviews, participants were asked to recall their grades for each completed unit, which they did with varying levels of certainty and specificity. When aggregated, these self-reports suggest that the majority of student participants typically achieved “distinction” level grades, with the remainder averaging at the “credit” level^2^. They were recruited via posters placed around university campuses and snowballing.

#### Materials and procedure

Data were collected through semi-structured interviews conducted by a research assistant, and guided by a protocol which began by asking participants about the nature of the research methods and statistics units they had taken, and their reflections on those units. They were then directed to a set of brief research vignettes (reproduced in Appendix A in Supplementary Material), prompted to imagine they were the researcher depicted in each, and asked to describe how they would determine appropriate statistics to use. Note that participants were not asked to actually identify a test or procedure (although many did), but rather describe the process or processes they would use to identify one. Following exploration of the vignettes, participants were asked to articulate the reasoning behind the processes they described, and identify processes that others may use in similar situations. Participants were then invited to describe their previous experiences with scenarios like those presented in the vignettes, and prompted to consider the role that an ability to solve such scenarios (or knowledge of an effective process for solving them) plays in a psychology graduate's repertoire of skills. Finally, the interviews concluded by asking participants to describe a tool or resource that they could use to help them approach and solve scenarios like those depicted in the vignettes. The full semi-structured interview protocol is reproduced in Appendix B in Supplementary Material.

Eight interviews were conducted face-to-face, with the final interview conducted via Skype. Each lasted between 30 and 50 min, and was audio recorded for later transcription. Prior to each interview, participants were presented with a participant information sheet, and were given the opportunity to have any questions answered. Face-to-face participants were then asked to sign a consent form, whilst the Skype participant was asked to indicate verbal consent after the consent form had been read aloud by the interviewer. At the conclusion of each interview, and before the recording device was turned off, participants were asked to verbally re-confirm consent, as recommended by Davis et al. (2004).

### Data preparation and analysis

The audio recordings were transcribed verbatim, and the transcripts were then independently verified for accuracy. The transcripts were imported into NVivo 10, and analyzed following the stages of thematic analysis outlined by Braun and Clarke (2006). Firstly, each transcript was read and re-read, while noting down initial impressions and ideas. Following this initial familiarization stage, the data were systematically coded in a line-by-line fashion. Codes were then collated into potential themes, which were continually reviewed and refined with reference to the source data and in consultation with team members, colleagues and the research literature. In the final stages of analysis, the themes were defined, and vivid data extracts relating to each were noted for inclusion in this paper.

### Findings

Several themes emerged from analysis of the student interview data. Firstly, students overwhelmingly found statistics to be challenging, yet acknowledged their importance for success in a range of different contexts. This is reflected in the theme, “*statistics are challenging, but important.”* On the whole, they found identifying appropriate statistical tests for the research vignettes particularly difficult, which resulted in embarrassment for some participants. Many struggled to describe a coherent strategy for approaching the vignettes, however some recognized that approaching them in a coherent and systematic way is possible, and tended to reflect on the utility of flow-charts and decision-trees they had encountered in their studies. These findings are captured by the themes of “*statistical selection falls outside the comfort zone,”* and “*a tenuous grasp on an elusive process.”* The students offered a variety of suggestions when prompted to consider the format and features of “*an ‘ideal’ statistical decision making aid.”* Each of these themes is elaborated on in the following sections.

#### Statistics are challenging, but important

Some students indicated that they did not expect to be taught research methods and statistics when they started their psychology degrees (“*it was a bit of a shock initially*,” “*we were so underprepared*”). Others entered the degree with negative expectations about these subjects (“*you hear about statistics before you start psychology and you hear that that's the main reason people drop out*”). They found their early experiences with the subject matter challenging, reporting that there was a lot of “*new”* and “*difficult*” material to learn, and that they sometimes felt “*stressed*,” “*nervous*,” “*confused*,” “*overwhelmed*,” “*overloaded*,” or “*lost*.” However, they took some console from knowing that others shared these experiences:

*Everyone's in the same boat …knowing at the very start no one knows what they are doing and everyone feeling a bit lost, it helps you feel like, ah well, I'm not the only one that is having trouble with this*.

Many students reported lacking confidence in their abilities (“*I'm just useless at this side of things*”), and that they were not “math people.” For example, one fourth year student explained, “*I'm a words person not a numbers person, so I was really stressed about doing statistics at uni*.” One particular source of anxiety was an exaggerated concern over the consequences of making mistakes:

*Having to figure out what test I was going to use …and still thinking, okay I'm certain, but I'm also a bit unsure. If I pick the wrong test [it will have] a domino effect. Everything else isn't going to work. It …made me feel so nervous*.

With experience, the subject matter became more manageable, and students' confidence grew. For example, one third year student remarked that, “*once you've got your foot in the door you can just sort of push through and it's easy*.” Having “pushed through the door,” research methods and statistics became considerably more enjoyable and rewarding:

I loved it once I understood it. But just having to go through the stress of trying to understand…getting [tutor] to explain it to me, going over the notes and trying to understand it, getting friends to explain it to me, that was very stressful and that's the part that I just didn't like…But once you actually get a grip on it…I love it!

Despite the challenging nature of the subject matter, students consistently acknowledged the value of research methods and statistics to the development of critical thinking (“*you can question more things, like under what circumstances did they come to that conclusion?*”), to success in their courses, and to competence as future researchers and evidence-based practitioners.

I'm excited to do honors; to do all the data analysis by myself, and I get to find out things and interpret the numbers. It's like bringing numbers to life, so that's exciting!

*It's important because…psychological research drives all other psychology. It's what forms and guides what every other psychologist will do and practice…or it should do anyway*.

#### Statistical selection falls outside the comfort zone

Although we did not ask participants to attempt actually solving the research vignettes, this was the first instinct for many. Most found the task too difficult. They were apologetic and expressed embarrassment at being unable to successfully complete a task they felt they ought to be able to complete:

I wish I could have done a bit better for you…

*[Interviewer: Do you think that being able to solve problems like these is an important skill for psychology graduates?] Of course, it's a bit embarrassing that I can't do it too well*.

However, there was a smaller cohort who jumped straight to a statistic. Occasionally, they did so correctly. Usually though, it was with an unwarranted level of confidence. For example, when presented with a vignette depicting the relationship between two binary variables, a student mid-way through his third year of study answered, “*so it would be a paired samples t-test. Yep that's right. Yep, pretty sure.”*

#### A tenuous grasp on an elusive process

When prompted to think about the process of selecting a statistic (rather than actually identifying one), students typically struggled. This was the case even for students who had completed several research methods and statistics units:

*[Interviewer: So how would that help you to decide which statistical test to use?] Um see I, see I'm thinking you'd probably want to…I'm sorry. I can't remember, sorry*.

The processes they described tended to be haphazard and inefficient, and included looking for (potentially misleading) clues in the wording of the vignettes (“*these scenarios are always worded in certain ways”*), searching through textbooks, lecture notes (“*I would probably just look at …every single test that I've learned about”*), the world-wide-web and previous research addressing similar research questions (“*you've got the journals and things like.…copy their methodology”*). They also reported relying on memory and prior experience or the advice of friends and teachers (“*you could ask your lecturers…‘Hey, I'm doing this assignment; what do you reckon I should use?”’*). Some suggested starting by entering their data into a spreadsheet, following a process of elimination, using mnemonic devices or simply guessing:

I kinda try and I guess. I don't know, they're never set in stone, I just kinda think like, ‘oh that's probably that one.’

Some students did recognize that a systematic decision making process could be followed: “*you go through checklists in your head*.” However, none could identify every factor requiring consideration before an appropriate statistic can be identified. Most also identified irrelevant factors. For example, in the following quote, a fourth year student correctly recognized that she needs to identify the independent and dependent variables (IV and DV), as well as the number of groups being compared. However, she did not consider the measurement levels of the variables (although a nominal IV is implied by her reference to “groups”). Furthermore, she identifies causality as an issue warranting consideration. The appropriateness of causal inference is almost entirely determined by research design, and has very little to do with choice of statistic:

Figure out the variables, the IV, DV I guess. How many groups there are, and what kind of, is it a correlational relationship? …Is it cause and effect?

Those students who recognized a process tended to refer to graphic organizers or decision trees in their statistics textbooks. They reported that such aids facilitated statistical decision making:

*The tree! The wonderful tree! It is very simple, easy to use and it pretty much points you right into the analysis that you need to do*.

#### An “ideal” statistical decision making aid

Knowing that students find selecting appropriate statistics challenging, we asked those in our sample to explore what might make the process easier. Many turned first to their instructors, who simultaneously helped students master conceptual issues and overcome their hesitation around statistics. When prompted to think about resources they could use independently, technologically based aids were commonly considered:

*If you had a website [which] just [asked] how many variables do you have? You know, how many dependent? How many independent? What are you looking at? What are you comparing to what? And it just tells you this is the test you use*.

This idea of a digital decision tree, which focuses the user on a sequence of key decision points before providing a solution was raised often. However, not all students had a preference for digital, with one remaking that she's prefer something in a hard copy format, “*because I can write into it like different things.”* Other features of an “ideal” aid included simplicity, accessibility, and multiple levels of depth, as illustrated in the following quotes:

*Once you've got the ease-of-use down and you can easily access it, and it tells you exactly what you need to do, I think that's probably all you need really, because once you set it up you can be autonomous and you can self-direct to what you should be doing*.

*It would be a merge between a super simple tree diagram, but then [a] step-by-step SPSS guide book [and] behind all that a really detailed kind of book …something that comes in three steps: simple, medium and really detailed*.

Additionally, students were aware of how the content they access on the world-wide-web is of variable quality, and expressed a preference for content endorsed by recognized “experts,” such as “*a psychologist…someone who knows it's going to be useful for other psychologists*,” or “*some Australian government agency*.” And finally, an “ideal” aid would contain engaging examples and links to other reputable resources:

*Just use like real life examples…like something to do with a person and a situation, instead of saying a group of researchers want to research rats and blah blah*.

*If there was a way to find more resources…a way to link you with more critical approaches to some statistical tools*.

### Summary

In the first phase of this study, undergraduate psychology students found our discipline's emphasis on research methods and statistics unexpected, and they approached these subjects with apprehension. They found statistics particularly challenging, but appreciated their importance to success in a range of contexts. Making statistical decisions fell outside the comfort zones of most students, which caused some embarrassment. They had a tenuous grasp on the decision making process, but recognized resources and aids that could guide them through it. When asked to consider the format and features of an “ideal” aid, they expressed a preference for an accessible, comprehensive, and reputable resource that follows a basic decision tree logic.

In the second phase of this study, we turn our attention to the statistical decision making approaches used by psychology academics with particular expertise in conducting research and/or research methods instruction. We also explore their perspectives on the challenges students face when required to choose appropriate statistical tests and procedures, as well as their thoughts about resources that could facilitate this process.

## Phase two: Academics' decision making

### Methods

#### Participants

The second phase participants were 10 psychology academics (five female) with appointment levels ranging from lecturer to professor (with a median level of senior lecturer). Six had traditional teaching and research roles, and the remainder were research focused. All were PhD qualified, research active, publishing several papers per year, and supervising research students at the level of honors and above. They predominantly identified as quantitative researchers, although some also used qualitative methods, dependent on the topic of investigation. Half had also coordinated at least one research methods and statistics unit during at least two of the preceding three years. The academic participants were recruited via individual emails, either directly from the first author's professional network, or via colleagues. They were not financially or otherwise compensated for their participation.

#### Materials, procedure, data preparation, and analysis

Data were collected through semi-structured interviews conducted by the second author, who did not have a dual role (e.g., as a colleague) with any of the participants. Eight were conducted face-to-face, with the remainder conducted via Skype. As in phase one, all interviews were audio-recorded, following the procedures for obtaining consent described previously. They were guided by protocols (see Appendices C,D in Supplementary Material) that began by querying the functions that statistics play in psychological research and the psychology curriculum. Participants were then directed to the set of research vignettes (presented in Appendix A in Supplementary Material), and asked to describe and explain the process they would use to identify an appropriate statistical test or procedure for each. They were then invited to describe their previous experiences with similar vignettes, and the role that being able to solve them plays in a psychology graduate's repertoire of skills. We then described to participants what we had observed when presenting the vignettes to students in phase one of the study. Specifically, we explained that most of the students struggled to articulate a coherent process, and when they attempted to solve the scenarios they tended to do so incorrectly. We then asked participants why they thought the students found this task so difficult. Finally, participants were asked to describe a tool or resource that students could use to help them approach and solve scenarios like those depicted in the vignettes. Following the interviews, the audio recordings were transcribed, and the transcriptions were analyzed using the techniques described previously.

### Findings

Like the students, the academics in the sample also described the importance of statistics, both to their work and the discipline of psychology. They saw “*statistics as a tool*” (amongst several) of research. From their vantage point, the academics also reflected on the nature and value of training in statistics, which they linked primarily to the development of critical thinking and evidence-based practice. This is captured in the theme, “*statistical training underpins competence*.” When prompted to describe the factors that influence their statistical choices, the academics described a complex, nuanced and iterative “process,” during which many factors warrant consideration. Some of these factors emerge from the research question and design, whilst others are linked to characteristics of the researcher and broader contextual considerations. These findings are reflected in the theme, “*decision making is a multifaceted process.”* The academic participants recognized that “*students find statistical selection challenging*,” and this knowledge informed their “*pedagogic practices*.” Finally, they described “*an ‘ideal’ statistical decision making aid*” which shared many of the features identified by the students, but placed a greater emphasis on “the process” rather than “the answer.” Each of these findings is elaborated in the sections that follow.

#### Statistics as a tool

When asked about the role that statistics play in their work, the academics used terms such as “*central*” and “*vital*,” and suggested that research would be “*pointless*” or “*nothing*” without statistics. However, despite being necessary to quantitative research, being a quantitative researcher requires much more than just knowledge of statistics. To illustrate this point, the “statistics as a tool” metaphor was regularly evoked. For example, “*the way I describe it to students – it's like if you're a tradie or a carpenter, then statistics are your hammer.”* Furthermore, rather than assuming a primary role in the research process, statistics are subservient to the research question and design:

*The important thing about research, as far as I'm concerned, is not the statistics. That's a tool that you use at the very end in order to answer the question. The important thing in my book is the questions that you're dealing with, that you develop, and the experimental designs that you then use in order to answer your questions*.

In other words, the statistics “fall out” of the design, and the design is a logical consequence of the research question. Or, to quote one of the senior academics in the sample, “*we have a question, we come up with a method of testing it, and we test it and then we move on from there. We get the answer and that the answer is given to us by statistics*.” It is not (or should not be) the reverse:

*I don't look at it like, ‘well I like this statistic, so, I'm gonna design all kinds of studies that I can use this statistic for, or this method for’. I try and look at it the other way around, which is what you're supposed to do*.

#### Statistical training underpins competence

Participants saw the role that statistics play in psychology curricula as multifaceted, and that a rigorous background in quantitative methods can distinguish the psychology graduate from graduates of other disciplines, (“*that's what makes psychologists or psychology graduates cool and different*”). While noting that statistical literacy was a necessary precondition for conducting research, they saw the primary purpose of statistical training as tied to the competent consumption (and evaluation) of research literature and the development of critical thinking skills:

*I do think it's a very central skill that they should be able to come out and go, ‘Okay. Well, I can read this paper and think they've done the appropriate analysis,’ and not have to rely on conclusions the authors have drawn…You're sort of critically consuming information rather than just taking what you're told*.

Participants also saw training in research methods and statistics as providing a general framework for applied problem solving: “*I think that approaching complex social problems in general requires you to have an understanding of multivariate and quantitative statistics. So it makes you a more informed citizen*.” Furthermore, the ability to understand and evaluate research literature and solve problems were widely regarded as necessary pre-requisites for evidence based practice: “*We base our profession on the scientist-practitioner model, so the evidence base is very important and statistics are really the – what we use to establish that evidence base*.” However, this sentiment was not universal, with one participant commenting that, “*I'm not really aware of any data which suggests that their statistical expertize is associated with better performance as a clinician…Not everyone needs as much [training in statistics and research methods].”*

Despite generally recognizing their importance, some participants noted that we do not do a good job of communicating this importance to students, which may be linked to students often only appreciating the relevance of statistics and research methods in hindsight:

I don't think the reason we include them [statistics] in psych is ever made very clear to students

The feedback I get from students is often delayed…They come back a year later and say, ‘thank you, I really enjoyed that. Now I understand it.’ But it's a shame. I wish they would have had that eureka moment a bit earlier …

#### Decision making is a multifaceted process

When prompted to explicate the factors influencing analytic choices, participants described a complex, nuanced and iterative “process,” during which many issues warrant consideration:

Often there are a number of different ways to answer a question and which one's appropriate depends on the current state of the literature, obviously the data that you've collected, what it is you want to get out of it, where it's going to be published…

This process begins with “*the question*” and design, followed by the nature of the variables in the study. In fact, the prevailing attitude was that, without a clear research question and intent in mind, any discussion of statistics was premature. For example, when asked about how he would respond to a student who had research ideas, but was uncertain about the appropriate statistics, one participant stated, “*I would tell them that they shouldn't worry about stats; they should worry about the questions that they have, how they can operationalize the question, put it into a research design that will give them an answer, and then we'll worry about the stats later.”* However, while “*jumping*” into statistics too soon was regarded as poor practice, so was leaving the development of an analytic plan too long. Doing so can prove costly, as illustrated in the reflections of one senior research focused academic:

For one of the studies for my PhD I collected a load of data and then realized it actually wasn't analyzable in SPSS …And that's where I started realizing the importance of knowing what you're doing before you start, and not collecting data and then saying, ‘well, how will I analyze this?’

When developing an analytic plan, participants most commonly looked to aspects of the study. However, personal characteristics and contextual factors can also play a role in the decision making process.

##### Characteristics of the study

Having a clear understanding of the purpose and design of the study as well as the number and nature of variables were recognized as essential to being able to select an appropriate statistic. For example, when presented with the second scenario in Appendix A in Supplementary Material, an experienced research methods instructor explained:

*I see a between groups three level IV. And then I see a between groups two level IV. So I'm thinking a two by three factorial design. And I'm seeing this repeated measures …So at this point I can see there's a choice between - like the way it's written implies that the dependent measure is an average over five trials. So that's a 2* × *3 between groups design. Of course, you could look at it as a three way mixed ANOVA with ‘trial’ as a third factor, which allows you to look at trajectories of learning. So I'm thinking if I'm writing for a journal, a learning journal, I'm pretty sure that it would be a three way mixed design. As it's presented here thought it looks like a two by three between groups design*.

Participants also noted that consideration should be given to alternative options in the event that analytic plans require modification due to, for example, violated assumptions. The importance of considering Type 1 and Type 2 error rates, statistical power, and the directionality of hypotheses during the decision making process were also discussed. Notably, participants actively considered viable alternatives, and weighed up the benefits and challenges associated with different decisions. This was particularly evident when discussing the mentoring of junior researchers:

*Usually I will try and elicit their ideas first, and then pose some questions if I think there are other options, and ask whether they'd considered them. And if not, why not. Or if they had considered them, but decided on an alternative method, discuss why that is*.

There was also a degree of tension between what could be considered “ideal,” and what is realistic or possible. As explained by one of the instructors, “*there's quite a few different ways to actually do things, of varying levels of effectiveness, and depending on the resources that you have.”*

##### Personal factors

Participants expressed an element of personal preference when considering appropriate analytical strategies (“*I'm not a fan of mixed ANOVAs. I much prefer to go through with repeated measures ANOVAs…”*), although it was recognized that such an approach does not reflect “*best practice*.” There was also some tension between a desire to prove competence and an appreciation that the “best” technique is not necessarily the most complex:

There is something nice about really complex designs and really complex analyses that tend to stun people into thinking, ‘you know what you're talking about!’

*I tend to err on the side of you use the technique that's appropriate, not the fanciest one. So there's something to be said for if a t-test answers your question, use a t-test. Like there's no need to get all fancy just for the sake of it*.

##### Contextual factors

It was observed by academic participants that research is not conducted in a vacuum, and that there are factors outside the researcher's immediate control which influence the statistical decisions they make. The first of these is the intended audience: “*What people need to realize is that the choice of analysis is on par with choice of audience…[and] sometimes you have to do different analyses for different audiences*.” As reviewers and journal editors are frequently gatekeepers between researchers and their broader audiences, their opinions were given particular weight: “*Then you get a reviewer who has their own preference on the type of statistics they would like to be used, so you have to revise it*.” At times, these opinions were seen as useful, and helped shape future decision making. At other times, they could be an impediment to progress:

*I was always taught that if you're testing mediation, you should use Baron and Kenny's model which is now, indeed, 20 years out-of-date, and there are whole books on much better ways of doing it. And the only way I came across that was when I submitted a paper with mediation and one of the reviewers said, ‘yeah, this is okay, but there's much more sophisticated and better ways of testing that’. It put me into touch with a whole literature which I now – anytime I'm testing mediation, we use those*.

*And what I have experienced this last year, actually, is that I did use different statistical methods working with [a statistical consultant]…And because they were different, they were met with – reviewers didn't like it. They didn't like things that they didn't know. So you'd have to explain it, and they thought that you were trying to trip them up or trick them to get something*.

Participants also made regular reference to how shifting discipline practices (and what is considered “best practice”) can influence decision making. For example, one participant described how she used simple regression techniques in her PhD. Yet, if she was examining a current PhD in which the same techniques were used, she would say “*no way, go back and do something much, much better*.” Furthermore, although best practice guides decision making, what defines best practice is often quite opaque:

*There is uncertainty …because there's no black and white. It's not really that kind of field. So you might find one article that said, ‘breaking the assumption is okay under these circumstances. You can get away with it.’ And in other circumstances you can't. So you often get contradictory messages*.

The preceding quote indicates that there may be a range of “best practices,” and what is ultimately acceptable depends both on the technique applied, as well as its justification:

*With my graduate students, a lot of what I'm teaching is ‘yes there are some fundamentals, but once you get beyond that it's about being able to determine the appropriate technique for your question and your data and then be able to justify that decision knowing that you'll send it out for review and people will disagree with you’*.

Finally, beyond an aspiration toward best practice, participants also indicated a desire to avoid (or be seen to avoid) poor practice. The poor statistical practices most commonly cited centered on “fishing” for effects and their subsequent misrepresentation in published work:

*If you're just doing post hoc analysis, but pretending that it was a priori, then you get – I've seen it at conferences; students claiming they did a mediated moderation on one thing and then moderated mediation on the other. And you kind of go, ‘there's no way that was a priori. You did not go into the research with that plan!’ If you do enough statistical tests and you don't report them, and you don't do Bonferroni corrections, then you run the risk that something is going to be significant, just because*.

#### Students find statistical selection challenging

Aside from a small cohort of particularly capable students, it was widely recognized by the academic participants that many students find research methods and statistics challenging sections of a psychology degree. When we described the outcomes of presenting the research vignettes to the student sample, and asked academic participants why they thought the majority of students struggled with them, a range of possibilities were suggested. Some of these appeared to be attitudes or dispositions that students brought to the degree or developed over time, whereas others reflected characteristics of the teaching methods and materials commonly used in undergraduate psychology courses.

##### Student characteristics

Participants perceived that the reality of a psychology degree is often inconsistent with students' expectations on entering the course. This could be because psychology “*doesn't sound like a course that requires a lot of statistics*.” They also noted that many students approach statistics with anxiety, lack confidence in their statistical abilities, are disinterested in research methods and statistics, or do not see their relevance to their future professional lives:

*Students are scared of statistics. And therefore they get a bit of a mental block, I think, and convince themselves they don't know how to answer the question*.

*It's perceived as another class they don't like, that they don't perceive is relevant, that they don't understand – It's like math at school, ‘when am I ever going to use this?’ Because students coming in are all gong to be clinical psychologists and we know clinical psychologists never use numbers* <*laughs*>*!*

##### Course characteristics

Academic participants highlighted both implicit and explicit characteristics of the research methods and statistics curriculum which may hinder, rather than support students' skill development. For example, one participant described the discipline's tendency to “*fetishize*” statistics, and how this value is communicated to students:

“*There's an element of elitism. If we make it seem really hard and difficult to get into and make it really opaque, we're shoring up this idea that stats is for the hard men and the real - we can sort the men from the boys amongst the students and also amongst everyone else of us too.”*

Others spoke of teaching approaches which tend to compartmentalize content, which is stripped of context when presented to students:

*It was very much pigeon-holed. So it was very much this week we're talking about ANOVA; this week, we're talking about regression; this week, we're talking about something else. So there really wasn't that opportunity to make a decision about which one is which. It was just, ‘this is what you're doing’*.

Overwhelmingly though, participants ascribed the difficulties students have with statistical decision making to teaching methods which don't engage students in regular decision making opportunities from early in the course (“*there just isn't enough exposure to that sequence of thought and planning*”), and don't regularly reinforce the relevance of statistics. It was considered that both these aims could be achieved by engaging students in the full research “process.” To participants, this process begins with a substantive research question, works through key issues tied to design and analysis, and concludes with clear implications or, to quote one instructor, an answer to the question, “*what does this shit actually mean?”*

*Showing that it's not necessarily about numbers but about answering questions might help with some of the – and putting it into that context, and putting it into the context of a research problem and not a math problem - I think, it can help as well*.

Answering questions of substantive interest was seen as vital. Furthermore, failing to achieve this aim may promote disinterest, disengagement, and apathy.

…*as soon as it's a question that you wanna know the answer to, it's like …it suddenly becomes relevant and important*.

#### Pedagogic practices

Recognizing that statistical decision making is an area that students find challenging, participants employed a number of techniques to encourage and support their efforts. This tended to occur in the context of either small-group/individual research supervision sessions or lab group meetings. Firstly, questioning was used to guide students “*through the process*.”

*I use a lot of questioning and I'm just thinking about one student that I spoke to just last week who put point blank to me, she said, ‘oh, we'll be using [multiple] regression to answer this question,’ and I immediately sort of flicked it back on her and said, ‘but how are you measuring your DV?’ – which was dichotomous. So in asking that question, she was able to go, ‘oh hang on a minute…that data is not appropriate for what I just said’*.

The process involves considering design and statistical issues concurrently, and in the context of the research question or objective:

*I ask them to draw out the design of an experiment, say, and they might suggest some stats at the end. And then, I ask them how that addresses the question or questions [they] want to get to*.

It also involves consideration and evaluation of different options before making decisions, and collaboration and consultation is encouraged:

…*try and present the different options…what are the pros and cons of each in this case, and then weigh those and come to a decision. I think you kind of need to let them go through the process*.

#### An “ideal” statistical decision making aid

Academic participants suggested characteristics for a tool or resource that students could make use of to independently identify appropriate statistics for various circumstances. First, the resource should be accessible (in terms of ease and cost of availability), and step users through a sequence of questions or decisions which must be addressed to arrive at an actionable outcome. Terms like “*flow-chart*” and “*decision-tree*” were used commonly.

*It is a question and answer flow-chart kind of situation. Is it relationships or differences? …how many variables; categorical or continuous? The answers to each of those questions would lead you to the correct [statistical analysis]*.

*It seems like if there was some sort of decision tree …It would make sense to have some sort of app or something …easily accessible online or on your phone or whatever, where you can plug in and go through a step-by-step process*.

If questions or decision points are presented sequentially, the user is forced to engage with each step in “*the process”* and can thus be “*train[ed] …to ask the important questions.”* The longer term objective of such a resource should not be reliance, but rather a transition toward greater autonomy and flexibility:

[After using the resource for a period of time, the user should ideally be able to] turn it off or turn the book over and then you give them another problem and see, well can they now - are they now able to - even if they can't get to the right answer, are they now trying to figure out? ‘Well, what am I trying to do? How many groups and what am I - what's my IV, what's my DV, do I have more than one IV, what's the level of measurement?’

Participants also noted that understanding key terms (or having the ability to quickly look them up) is essential to being able to use such a resource effectively (“*you need to know what a covariate is, what the IVs and the DVs, what this actually means*”). Finally, they acknowledged that, realistically, such a resource is never going to capture all the nuances in statistical decision making, but may be useful within the broader discussion:

*If you try to reduce it to a few basic principles then you're missing critical questions, like ‘what is the hypothesis’ and ‘what is the audience’? It's really much better if it's a consultative process with an advisor and/or with other [students]. I don't think people should work independently necessarily. I think that there's a lot of virtue in consulting with people in the design phase of the project*.

### Summary

In this study's second phase, the academics saw statistics as one of several tools available to the researcher; a tool that is vital to the conduct of most research, but subservient to the research question and design. They acknowledged the role that statistics training plays in the development of research skills, but saw its primary role as nurturing the development of critical thinking and evidenced-based practice. The academics described choosing an appropriate statistic as a complex, nuanced, and iterative process, during which consideration should be paid to multiple contextual factors in addition to the characteristics of the study. They were sensitive to the challenges that many students experience when making statistical decisions, which they attributed partially to how research methods and statistics are commonly taught. This sensitivity was reflected in their pedagogic practices. The “ideal” statistical decision making aid the academics described shared many of the features identified by the student participants, although greater emphasis was placed on “the process” than “the answer.”

## Discussion

The first aim of this research was to explore the strategies that psychology students and academics use to select statistical tests. We probed these strategies in semi-structured interviews, in which participants were encouraged to discuss how they would approach each of a series of short research vignettes. Our findings indicate a number of key differences between how these two groups approach statistical decision making.

For the students in our sample, being required to make such decisions pushed them outside their comfort zones, resulting in either apologetic discomfort, or instinctual selections that were frequently incorrect. This finding is not surprising given the body of literature demonstrating that most students find statistics generally (Garfield and Ben-Zvi, 2007; Murtonen et al., 2008), and statistical decision making specifically (Ware and Chastain, 1991; Gardner and Hudson, 1999) to be difficult. Their ability to even describe the process of selecting a test was limited, and relied heavily on the use of strategies unlikely to produce optimal outcomes. These included searching through textbooks, lecture notes, and the world-wide-web, relying on memory and prior experience, turning to the advice of friends or teachers, and looking for clues in the wording or structure of the vignettes. A number of these strategies were also suggested or displayed by the students in Gardner and Hudson's (1999) research, who were particularly prone to misinterpreting research questions, and being mislead by key words and data presentations formats. Like those in Gardner and Hudson's research, the students in our sample were reasonably far into their degrees and were, on average, in their third year of study.

There were a minority of students who recognized that a systematic decision making process could be used to approach and “solve” the research vignettes. However, none were able to identify all the factors in the vignettes that would require consideration before appropriate statistics could be identified. Furthermore, these students had a tendency to also identify features of the vignettes which were irrelevant to the task at hand. Again, these findings are broadly consistent with Gardner and Hudson (1999), whose students often failed to take the nature of data (e.g., nominal, ordinal etc.) into consideration when making statistical decisions.

By way of contrast, the psychology academics described selecting appropriate statistics as a complex, nuanced and iterative process, embedded within the broader process of conducting research. They demonstrated how during statistical decision making, consideration ought to be paid to multiple contextual factors (e.g., the intended audience, prevailing discipline trends and practices etc.), in addition to the intent and design of the study itself. These experts were able to suggest appropriate statistical analyses for each vignette with ease, but were often reluctant to do so without understanding the purpose of the research, or having an opportunity to explore alternative possibilities. This behavior is suggestive of “structural awareness,” which is an ability to see past the surface features of a problem, and focus on its structural characteristics and the relations between them (Quilici and Mayer, 2002)^3^. It is a characteristic common to “expert” problem solvers across a wide range of specialized domains (Rabinowitz and Hogan, 2008).

Previous research suggests that structural awareness tends to develop naturally with experience (Rabinowitz and Hogan, 2008). In the Australian context, opportunities to engage in statistical decision making are limited prior to fourth year when, under individual supervision, psychology students embark on their first major research project. During this intensive research internship, expert supervisors model the statistical decision making process, and use a range of techniques to promote its development in students. Students in earlier years are largely reliant on lectures, laboratories, and tutorials to develop their research skills, and alternative methods of teaching statistical test selection, which are not reliant on individual supervision, are required for these years.

Our recommendation is to provide students with regular opportunities to engage in the statistical decision making process in the context of class research projects. It is widely recognized that scaffolded immersion in all aspects of the research process, from participation and/or data collection, through the development and testing of hypotheses, to the interpretation and reporting of findings, is a particularly effective way of teaching research skills (Bradstreet, 1996; Marek et al., 2004; Roberts and Allen, 2012, 2013; Earley, 2014; Stoloff et al., 2015). This point was echoed by the academic participants in the current research, who reflected on how embedding statistical decision making in a context of substantive interest, and providing opportunities to work with personally meaningful data promotes student engagement. As an example, in the first author's second year experimental methods and statistics unit, students participate in an experiment early in the semester, which forms the basis of a research report assessment. The topic varies from year to year, but typically involves studying a well established phenomenon in a contemporary context (e.g., the attractiveness stereotype on Facebook; or the Internet as a transactive memory source). In a series of class and homework exercises, students are required to develop one or two theoretically meaningful hypotheses, use the class generated data to test them, and then prepare an American Psychological Association (APA) style research report for assessment. The experiment is usually structured such that several meaningful hypotheses are possible, and testable using techniques taught in the unit (which include parametric and non-parametric tests for comparing independent and related groups). One of the key tasks in this process is the identification of an appropriate statistical test for each hypothesis. Of course, such class research projects need not be the exclusive domain of research methods and statistics units, and can also be deployed effectively to teach a wide range of subjects (e.g., Lutsky, 1986; Ragozzine, 2002).

The second aim of this research was to solicit psychology students' and academics' views on the nature of resources that could facilitate the statistical decision making process. The findings indicate that both groups support the development of a digital decision tree that is simple to use, easy to access, provides multiple levels of depth, and is endorsed by “experts.” The psychology academics also stressed the need for such a resource to function as a teaching tool, which engages students with each choice-point in the decision making process, rather than simply providing an “answer.” This is in contrast to some recent trends in statistics software development to automate the test selection process based on the characteristics of the user's data file (e.g., “Nonparametric Tests” in IBM SPSS; Wacharamanotham et al., 2015). In fact, such trends are antithetical to the views of the academics in our sample, who strongly believed that statistics should be considered concurrently with other design issues, and far before any data are collected.

Based partially on the findings of the current study, as well as existing literature on the efficacy of decision trees and mobile learning technologies, we have recently published StatHand (see https://stathand.net), a free cross-platform mobile application designed to support students through the statistical decision making process. This application, developed with the support of the Australian Government Office for Learning and Teaching, guides users through a series of annotated questions to ultimately offer them the guidance necessary to conduct a suitable statistical test, as well as interpret and report its results. A full discussion of StatHand is beyond the scope of this paper, but interested readers are referred to Allen et al. (under review). In this paper, we overview the rationale behind StatHand, describe the development process and feature set of the application, and provide guidelines for integrating its use into the research methods curriculum.

When interpreting the findings of this research, readers should give consideration to the usual caveats regarding small samples and the transferability of qualitative research findings. The nature of the task we asked of participants (i.e., to describe how they would identify a suitable statistic) also warrants some consideration. It is plausible that the apparent deftness with which the academics approached this task is at least partially a function of the nature of their work, in which we imagine they routinely practice the metacognition and self-reflection for which we probed^4^. By contrast, it is suspected that the students in the sample have less experience with such skills, and fewer daily opportunities to practice them. However, this is a matter requiring attention in future research. Future research should also focus on exploring theoretically driven strategies and resources that may facilitate the statistical decision making process, and speed up the development of selection skills and structural awareness. To date, work in this area has largely focused on involving students in concrete research projects (e.g., Kardash, 2000) or the use of decision trees (e.g., Carlson et al., 2005; and the current research). Future work should be methodologically rigorous, and based on experimental methods, rather than the non-experimental and quasi-experimental approaches so commonly utilized in teaching and learning research (Wilson-Doenges and Gurung, 2013).

In conclusion, this paper presents a qualitative exploration of the strategies psychology students and academics use to make statistical decisions. The students in our sample found this task challenging, and many struggled to describe a coherent strategy for choosing appropriate statistical tests for common research scenarios. Those who did recognize that such scenarios could be approached in a systematic fashion tended to reflect on the utility of decision trees they had encountered in their studies. Unlike the students, the academics described selecting appropriate statistics as a complex, nuanced, and iterative process, embedded within the broader process of conducting research. When both groups were asked to imagine tools or resources that could facilitate the statistical decision making process, they tended to describe digital technologies based on a decision-tree framework. To the academics in particular, it was important that such resources scaffold the development of independent decision making competence, and not strip the user of the learning opportunities inherent in working through the full research process.

## Author contributions

PA conceived and designed the study with the support of LR. KD conducted the second phase interviews. PA analyzed the data and lead the writing of this manuscript, both with support and contributions from LR and KD.

### Conflict of interest statement

Conflict of Interest Statement: The authors declare that the research was conducted in the absence of any commercial or financial relationships that could be construed as a potential conflict of interest.

## Footnotes

^1^In the Australian context, a “unit” refers to a single subject, typically taken alongside two or three others over a semester. The term is analogous to “course” in United States higher education parlance.

^2^A “credit” indicates a final mark between 60 and 69%, and a “distinction” ranges from 70 to 79%. For reference a “credit” is typically considered “average” in Australian undergraduate degrees.

^3^Despite this structural awareness, the findings suggest that some psychology research academics have preferred techniques, will at times select techniques based on what they can “sell” rather than current best practice, and are reluctant to be early adopters of new techniques. This “resistance” by substantive psychological researchers to changing statistical techniques and employing new advanced statistical techniques has previously been recognized in the research literature (Sharpe, 2013). It has been attributed to a combination of a lack of awareness of new statistical developments, inadequate statistical education, the failure of journal editors to act as catalysts for change, the pressure to “publish or perish,” and fear of deviating from normative statistical practices (Sharpe, 2013).

^4^As kindly noted by one of our reviewers, the apparent deftness with which the academic participants were able to explore possibilities and identify suitable statistics sits in contrast with our discipline's well known difficulties when it comes to interpreting such statistics (e.g., Cohen, 1994; Hoekstra et al., 2006, 2014; McGrath, 2011; Kline, 2013).
