# Supplementary material for: Difficult Decisions: A Qualitative Exploration of the Statistical Decision Making Process from the Perspectives of Psychology Students and Academics
Source: Front Psychol. 2016 Feb 16;7:188. doi: 10.3389/fpsyg.2016.00188 (PMC4754455; doi:10.3389/fpsyg.2016.00188)
Supplement: Supplementary file 1 [file DataSheet1.docx]

# Appendix A: Research vignettes

Note that scenario 2 was only presented to the academics.

## Scenario 1

You are interested in whether or not someone has a mortgage is related to whether they prefer to watch commercial or non-commercial television news programs.

## Scenario 2

You work in an animal laboratory, and have been asked to investigate whether rats can be ‘bred’ to perform well on a T-maze task. (This is a commonly used task requiring that a rat learn which features of the maze identify where food is located.) As a secondary consideration, you’ve been asked to look at whether performance is also influenced by the nature of the environment in which the rats were raised. You have access to a group of rats that have been selectively bred to perform exceptionally well on this task (the ‘bright’ rats), a group that have been selectively bred to perform poorly on the task (the ‘dull’ rats), and a group who were bred without regard for their maze performance (the ‘control’ rats). Furthermore, half of each group has been raised in an ‘enriched’ environment, whilst the other halves have been raised in an ‘impoverished’ environment. Performance on the T-maze is measured as the time that it takes for the rat to find the food, averaged over five trials. There is one trial every 48 hours, and testing begins when each rat is exactly 60-days old. Your objective is to find out if and how breeding and environment influence T-maze performance.

## Scenario 3

Your friend works in an animal laboratory, and has been asked to design a series of experiments to find out which nutritional supplements produce optimal cognitive performance in rats. He has lots of ideas about how to measure cognitive performance, has access to several different nutritional supplements, and has a good knowledge of experimental design. However, he is absolutely useless at statistics, and has turned to you for help!

## Scenario 4

You work at a university library, and have been tasked with finding out which students accrue the largest ‘overdue fines’. The head librarian has provided you with a data file that gives you the total amount of fines (in dollars) accrued by each borrower during the previous 12 months, along with a range of additional information (e.g., each borrower’s course of study, age, gender, number of items borrowed etc.). You’re fairly confident that you’ve heard about statistical techniques suitable for answering this kind of question at some point in the past, but can’t remember when or where.

# Appendix B: Interview protocol for student participants

1. I’d like to begin by asking you to describe the research methods and statistics units that you’ve taken in your course at XX University. Can you tell me about how many you’ve taken; about how they were structured; and about the things that you learned in each?
   - Prompt for both content and structure as necessary.
2. If you don’t mind me asking, what type of marks do you get in [units identified above]?
   - If hesitant, ask for a ‘ball park’ figure (e.g., “credits, distinctions?”).
3. Did you enjoy the [units identified above]?
   - Prompt for what was enjoyed, and why.
4. [If not covered under 3] What didn’t you enjoy about [units identified above]?
   - Prompt for why.
5. I’m going to show you four short research scenarios [see Appendix A]. I want you to try and pretend that you are the researcher in each scenario, and that you want to work out which statistical test you would use. I DON’T want you to actually try and work out which tests to use though! What I’d like you to do is explain to me HOW you would work out which tests to use. Describe to me, step by step, what you would do.
   - Prompt for the pieces of information that need to be extracted from the scenario.
   - Prompt for what the student would do after this information had been extracted.
6. Have you been taught how to solve problems like these in [units above]?
   - Prompt to describe/explain.
7. You said that you would use [XXX methods identified in 5] to solve problems like these. Can you explain why you would use these methods?
8. Can you think of any other methods that people might use to solve these sorts of problems?
   - Prompt for benefits and/or limitations of each.
9. Do you think that being able to solve problems like these is an important skill for graduates in [your course] to have?
   - Prompt for why or why not.
10. If there were a perfect tool or resource that you could go to when faced with a problem like these, what would it look like?
    - Would it be paper or electronic? How would you access it? How would it work?
11. Is there anything else you would like to tell me about research methods or statistics?

# Appendix C: Interview protocol for research focused academic participants

1. I’d like to begin by asking you to briefly outline your current role at the university, with specific focus on the main tasks that you undertake as researcher.
   - If necessary, prompt for typical number of publications per year.
2. Can you describe the role that statistics play in **your** research, as well as in the conduct of research more broadly?
3. I’m going to show you some short research scenarios [see Appendix A]. I want you to imagine that you are the researcher in each scenario, and that you need to work out which statistical test you would use. It’s not necessary to work out or tell me which tests you’d use. What I’d like you to do is explain to me HOW you would work out which test to use. Describe to me, step-by-step, what you would do.
   - Prompt for the pieces of information that need to be extracted from the scenario.
   - Prompt for what the researcher would do after this information had been extracted.
4. You said that you would use [XXX methods identified above] to identify appropriate statistical tests in situations like these. Can you explain why you would use these methods?
5. Have you always used these methods, or have your techniques changed over time?
   - If so, how?
6. Were you taught how to solve problems like these? Either as a student or earlier in your career?
   - Prompt to describe/explain.
7. Are you aware of other methods that researchers might use to identify the statistics they need to use to analyse their data?
   - Prompt to describe/explain where relevant.
8. When you supervise junior researchers (e.g., honours, HDR students), and they are unsure of which statistical analyses they should be using, how do you advise them?
9. We recently gave scenarios like those [Appendix A] to undergraduate psychology students, and asked them to describe the process they would use to identify an appropriate test or statistic for each. Most of them struggled to articulate a process, and the strategies that they could identify tended to be inefficient and haphazard. When they tried to actually solve the scenarios, they usually got them wrong (even the students in third and fourth year). Why do you think students find these tasks so difficult?
10. Do you think that being able to solve problems like these [Appendix A] is an important skill for psychology graduates to have?
    - Prompt for why or why not.
11. If there were some sort of tool or resource that we could provide students with to help them solve problems like these [Appendix A], what might it look like?
    - Would it be paper or electronic? How would you access it? How would it work?
12. Is there anything else you would like to tell me about research or statistics?

# Appendix D: Interview protocol for teaching/research academic participants who had coordinated at least one research methods and statistics unit during at least two of the preceding three years

1. I’d like to begin by asking you to briefly outline your current role at the university.
   - Prompt for overview of teaching responsibilities.
   - Prompt for overview of research responsibilities, including typical number of publications per year.
2. Why are research methods and statistics included in the undergraduate psychology curriculum?
3. I’m going to show you some short research scenarios [see Appendix A]. I want you to imagine that you are the researcher in each scenario, and that you need to work out which statistical test you would use. It’s not necessary to work out or tell me which tests you’d use. What I’d like you to do is explain to me HOW you would work out which test to use. Describe to me, step-by-step, what you would do.
   - Prompt for the pieces of information that need to be extracted from the scenario.
   - Prompt for what the researcher would do after this information had been extracted.
4. You said that you would use [XXX methods identified above] to identify appropriate statistical tests in situations like these. Can you explain why you would use these methods?
5. Have you always used these methods, or have your techniques changed over time?
   - If so, how?
6. Were you taught how to solve problems like these? Either as a student or earlier in your career?
   - Prompt to describe/explain.
7. In your teaching work (including research student supervision), do you teach students how to solve problems like these?
   - If so, how?
8. We recently gave scenarios like those [Appendix A] to undergraduate psychology students, and asked them to describe the process they would use to identify an appropriate test or statistic for each. Most of them struggled to articulate a process, and the strategies that they could identify tended to be inefficient and haphazard. When they tried to actually solve the scenarios, they usually got them wrong (even the students in third and fourth year). Why do you think students find these tasks so difficult?
9. Do you think that being able to solve problems like these [Appendix A] is an important skill for psychology graduates to have?
   - Prompt for why or why not.
10. If there were some sort of tool or resource that we could provide students with to help them solve problems like these [Appendix A], what might it look like?
    - Would it be paper or electronic? How would you access it? How would it work?
11. Is there anything else you would like to tell me about teaching research methods and statistics?
